# Genotype by random environmental interactions gives an advantage to non-favored minor alleles

**DOI:** 10.1038/s41598-017-05375-0

**Published:** 2017-07-12

**Authors:** A. Mahdipour-Shirayeh, A. H. Darooneh, A. D. Long, N. L. Komarova, M. Kohandel

**Affiliations:** 1 Department of Applied Mathematics, University of Waterloo, Waterloo, ON N2L 3G1 Canada; 2 Department of Physics, University of Zanjan, P.O. Box 45196-313 Zanjan, Iran; 3 Department of Ecology and Evolutionary Biology, University of California, Irvine, CA 92697 USA; 4 Department of Mathematics, University of California, Irvine, CA 92697 USA

## Abstract

Fixation probability, the probability that the frequency of a newly arising mutation in a population will eventually reach unity, is a fundamental quantity in evolutionary genetics. Here we use a number of models (several versions of the Moran model and the haploid Wright-Fisher model) to examine fixation probabilities for a constant size population where the fitness is a random function of both allelic state and spatial position, despite neither allele being favored on average. The concept of fitness varying with respect to both genotype and environment is important in models of cancer initiation and progression, bacterial dynamics, and drug resistance. Under our model spatial heterogeneity redefines the notion of neutrality for a newly arising mutation, as such mutations fix at a higher rate than that predicted under neutrality. The increased fixation probability appears to be due to rare alleles having an advantage. The magnitude of this effect can be large, and is an increasing function of the spatial variance and skew in fitness. The effect is largest when the fitness values of the mutants and wild types are anti-correlated across environments. We discuss results for both a spatial ring geometry of cells (such as that of a colonic crypt), a 2D lattice and a mass-action (complete graph) arrangement.

## Introduction

Virtually every species displays variation of the level of DNA. All variants at some time in the past were a newly arising mutation at a frequency of 1/*N* in a haploid population. There is an extensive population genetics literature describing the fate of such mutations in populations (see e.g. refs 1–3). Newly arising unconditionally deleterious mutations are typically quickly eliminated (although it is well-known that weakly deleterious mutations can also persist or even fix), mutations whose impact on fitness is equivalent to the wild-type allele at a loci will fix with probability 1/*N*_*e*_ (where *N*_*e*_ is the effective population size of the species), and mutations with a fitness advantage of *s* will fix with approximate probability *s*.

The probability of fixation has been widely studied by population geneticists, physicists and mathematicians for almost a century, starting with the early works by Fisher^4^, Haldane^5^ and Wright^6^, culminating in the work of Kimura^7^. These models have been extended in a number of ways, which can be roughly divided into three approaches: Markov chain methods, branching processes, and diffusion approximations (see ref. 8 and the references therein). These fixation probabilities have been derived using several methods and are considered quite robust^9–18^. A “Moran process”^19^ is one of the models used to derive these fixation probabilities. Under this model at each time step in a population of constant size *N*, a random individual is chosen for death and is then replaced with the progeny of another individual chosen for reproduction. The probability to reproduce is proportional to the individual’s fitness. The order of the update (birth-death or death-birth) has been studied in refs 20 and 21.

An attractive feature of a Moran process is its analytical tractability; also, since each time step consists of one individual replacing another, it is relatively easy to incorporate a spatial aspect to evolution. The Moran model allows for a natural spatial extension, such as a ring or a spatial lattice. In such cases, only neighboring individuals compete with one another to reproduce. If fitness values are equal with respect to genotype, but perhaps vary spatially, under a Moran process the probability of a newly arising non-favored (or neutral) mutation fixing is still 1/*N*_*e*_ (see e.g. refs 22–25). What if instead of fitness being only a function of spatial location, it is additionally a function of genotype? Such genotype by environmental interactions (GEI) are likely quite common. Classic examples include plant size along an altitudinal transect in *Potentilla glandulosa* ^26^, the emergence of the carbonaria form of the peppered moth as a response to the industrial revolution^27^, adaptive melanism in pocket mice living on different colored substrates^28^, and a repeated reduction of armor plating in freshwater sticklebacks^29^.

Several studies have investigated the importance of environmental fluctuations in evolutionary dynamics. Lewontin and Cohen^30^ studied a growing population in a randomly varying environment where the growth rate is a random variable. Other studies concerning population growth under environmental noise include Frank and Slatkin^31^, Haccou and Iwasa^32^, Yoshimura and Jansen^33^, and Mustonen and Lässig^34^. There have also been many other investigations of the environmental alteration in population genetics^35–37^.

Furthermore, environmental effects and randomness in fitness have also been studied in the context of the speed of invasion dynamics^38^, environmental-induced plasticity^39, 40^, and also in game theoretical models^41–43^. Various evolutionary strategies were discussed in Patra and Klumpp^24^ to show how the spatial structure of the population and environmental alterations might be beneficial for higher phenotypically heterogeneous populations.

More recently, in ref. 44, the authors considered two different types of heterogeneity, one where individuals had regional fitness values (‘*partisan voter model*’) and the other where their random fitness independently and intrinsically changed for individuals. Using mean-field approximation, they compared the time to fixation for each of these two models and studied the population-size dependency of ultimate time to fixation for finite populations. Melbinger and Vergassola^45^ considered the effect of environmental alterations on the fitness of species. They showed that variability in the growth rates played an important role in neutral evolution and the fixation time was reduced in the presence of time dependent environmental fluctuations. In addition, Cvijović *et al*.^46^ found that temporal fluctuations in environmental conditions could influence the fate of mutation and subsequently the efficiency of natural selection. Recently, Dean *et al*.^47^ studies stochastic and continuous time approaches for the selection of a two-allele haploid model under random environment, and argued that with a zero mean selection coefficient, temporal environmental fluctuations increase genetic polymorphism, which is in contrast with the classical selection theory. Assuming a stochastically neutral condition for alleles with and without a genetic mutation, they showed that randomness in selection increased the fixation probability in comparison to neutral drift. The derived fixation probability was based on approximating the ratio of growth rates of the diverse genotypes.

Expanding on the previous work, in this paper we consider environmental-induced random effects on the dynamics of mutants. The main focus of our work is spatial fitness fluctuations (as opposed to temporal fluctuations considered in many of the studies listed above), and we specifically address the question of mutant fixation. Our most intriguing and counterintuitive finding is that if both mutant and wild type fitness values come from exactly the same distribution, minority mutants nonetheless enjoy a selective advantage (averaged over all realizations of fitness landscapes). This phenomenon holds for both death-birth and birth-death formulations of the Moran process as well as for the Haploid Wright Fisher process. We further expand our calculations to consider a variety of scenarios, including the influence of the higher moments of the fitness distribution under identical and different fitness probability distributions, as well as the role of correlations between the two fitness distributions.

## Method

The calculations for this study have been performed for several different stochastic update rules. Here we give a detailed description of one of these rules (the death-birth Moral process). Others (the birth-death Moran process and the haploid Wright-Fisher model) are described in the Supplement. Envisage the Moran process^19^, where in a constant population of asexually reproducing agents (or cells), at each update, a cell is removed and replaced with the offspring of another cell according to some rules. Which cell gets to go and which one reproduces is decided probabilistically. For example, suppose that death (removal) happens with the same probability for all, and reproduction is performed for one of the “neighboring” cells, with probabilities proportional to the cells’ fitness (see ref. 20 for other rules). The notion of neighborhood is defined by spatial interactions and here we will specifically focus on the mass-action case where the whole population belongs to a cell’s neighborhood, and on a circular arrangement where each cell has only two neighbors. Reproduction is assumed to be faithful, such that the offspring cell inherits the type of the parent cell. As the updates continue, the population size remains constant, and eventually with certainty the whole population will be replaced with the offspring of a single cell (and the corresponding type will “fixate” in the population).

Let us first assume that the fitness of all but one cell is equal to 1, and a single cell has a smaller fitness, r < 1 (we will refer to this cell as a mutant, and the rest of the cells as wild type cells). Not surprisingly, the mutant cell in this case will have a smaller fixation probability compared to any of the wild type cells. In fact, in the mass-action problem the probability of such disadvantageous mutant fixation, *P*_*N*_, is exponentially small in terms of the population size *N*, and (for large *N*) is given by (1/*r* − 1)*r* ^*N*^. In the opposite case of an advantageous mutant (*r* > 1), the probability of mutant fixation is larger than the probability of fixation of wild type cells and is given by 1 − 1/*r* for large population. Similar results also hold in the case of a circular geometry, where the probabilities of disadvantageous and advantageous mutant fixation are given respectively by 2*r* ^*N*−2^(1 − *r*)/(3 − *r*) and 2(*r* − 1)/(3*r* − 1)^14^. Not surprisingly, if the fitness of the mutant is exactly the same as the fitness of the rest of the cells (*r* = 1, a “neutral” mutant), the probability that the whole population is eventually replaced by the offspring of such a mutant is exactly the same as the probability of fixation of any other cell, and is equal 1/*N* (this result holds both for mass action and for circular geometry).

The simple situations described above are idealizations because, for example, they do not include the inhomogeneity of the environment. Let us suppose that the fitness of a cell depends not only on its type but also on the spatial location. We will assume that the fitness of a wild type cell at a given location is drawn from a fixed probability distribution. We further assume that the fitness of a mutant cell in a given location is also taken from exactly the same distribution. In other words, for a single realization of a Moran process we need to assign the fitness values of wild type and mutant cells for each location, and these values remain fixed throughout the realization. What is the probability of mutant fixation (starting with one mutant cell at a randomly chosen location) averaged over realizations? Simple intuition would suggest that it is 1/*N*, because the fitness values of the mutant are taken from the same distribution as those for the wild type, and the probability is averaged over all the possible realizations. We show that the answer 1/*N* however is only true for *N* ≤ 3. Starting from *N* = 4, the average probability of fixation, 〈*P*_*N*_〉, of mutant in such a random landscape is greater than 1/*N*. In fact, for relatively small values of *N* the function 〈*P*_*N*_〉*N* grows linearly with *N* and for larger population sizes it continues to grow but becomes logarithmic (to compare, 〈*P*_*N*_〉*N* = 1 in the absence of randomness). One should note that when the mutants are advantageous then 〈*P*_*N*_〉*N* grows linearly with *N* and the slope increases with the standard deviation of the fitness distribution. If the fitness of the wild type is constant (*r* = 1), but the fitness of a mutant cell $(\tilde{r})$ at a given location is selected from a fixed probability distribution, then the average fixation probability is negatively correlated with the variance of fitness distribution of mutants (for both advantageous and neutral mutants)^48^. This could be due to the fact that increments and decrements of fitness values compared to the background fitness do not have a symmetric effect on the fixation probability. For example, if *r* = 1 and $\tilde{r}=1\mp 0.5$, then the devastating effect of hitting the value $\tilde{r}=0.5$ cannot be compensated by a relatively mild advantage of hitting $\tilde{r}=1.5$.

Consider a circle with *N* = 3 individuals. We denote the fitness for the wild (mutant) type by *a*_*i*_(*b*_*i*_), *i* = 1, 2, 3. Further, *P*_*s*_, where *s* is a binary number of length 3, stands for the probability of mutant fixation starting with configuration s, where “1” stands for mutant and “0” for the wild type. Defining ${f}_{\alpha }^{\beta }:=\alpha /(\alpha +\beta )$, we obtain for the death-birth Moran process:

1 $$\begin{array}{rcl}3{P}_{100} & = & {P}_{110}\,{f}_{{a}_{3}}^{{b}_{1}}+{P}_{101}\,{f}_{{a}_{2}}^{{b}_{1}}+{P}_{100}(\,{f}_{{b}_{1}}^{{a}_{2}}+\,{f}_{{b}_{1}}^{{a}_{3}}),\\ 3{P}_{010} & = & {P}_{011}\,{f}_{{a}_{1}}^{{b}_{2}}+{P}_{110}\,{f}_{{a}_{3}}^{{b}_{2}}+{P}_{010}(\,{f}_{{b}_{2}}^{{a}_{1}}+\,{f}_{{b}_{2}}^{{a}_{3}}),\\ 3{P}_{001} & = & {P}_{101}\,{f}_{{a}_{2}}^{{b}_{3}}+\,{P}_{101}\,{f}_{{a}_{1}}^{{b}_{3}}+\,{P}_{001}(\,{f}_{{b}_{3}}^{{a}_{1}}+\,{f}_{{b}_{3}}^{{a}_{2}}),\\ 3{P}_{110} & = & 1+{P}_{100}\,{f}_{{b}_{1}}^{{a}_{3}}+{P}_{010}\,{f}_{{b}_{2}\,}^{{a}_{3}}+\,{P}_{110}(\,{f}_{{a}_{3}}^{{b}_{1}}+\,{f}_{{a}_{3}}^{{b}_{2}}),\\ 3{P}_{101} & = & 1+{P}_{100}\,{f}_{{b}_{1}}^{{a}_{2}}+{P}_{001}\,{f}_{{b}_{3}}^{{a}_{2}}+\,{P}_{101}(\,{f}_{{a}_{2}}^{{b}_{1}}+\,{f}_{{a}_{2}}^{{b}_{3}}),\\ 3{P}_{011} & = & 1+{P}_{010}\,{f}_{{b}_{2}}^{{a}_{1}}+{P}_{001}\,{f}_{{b}_{3}}^{{a}_{1}}+\,{P}_{110}(\,{f}_{{a}_{1}}^{{b}_{2}}+\,{f}_{{a}_{1}}^{{b}_{3}}).\end{array}$$

The solution to this system, averaged over all realizations of the fitness configurations for a given distribution is *P*_100_ = *P*_100_ = *P*_100_ = 1/3 and *P*_110_ = *P*_101_ = *P*_001_ = 2/3. This results in the fixation probability 〈*P*_3_〉 = 1/3, starting from one mutation. The above formulation can be generalized to larger *N*, but against expectations, the probability of fixation starting from one mutant for *N* ≥ 4 is greater than 1/*N*, and more generally, the probability of fixation starting from *i* < *N/2* mutants is greater than *i/N*. The linear algebraic system can be solved exactly for relatively small values of *N*. For larger *N*, we perform numerical simulations of the Moran model (the results are verified by solving the corresponding Kolmogorov system of equations for 10,000 realizations). The birth-death formulation of the Moran process and the haploid Wright-Fisher model (see the Supplement for details) reveal similar patterns. In these cases, the probability of a mutant to fixate starting from *i* < *N/2* mutants is greater than *i/N*, even for *N* = *3* (and for *N* > *3*).

## Results and Discussion

**Figure 1:**
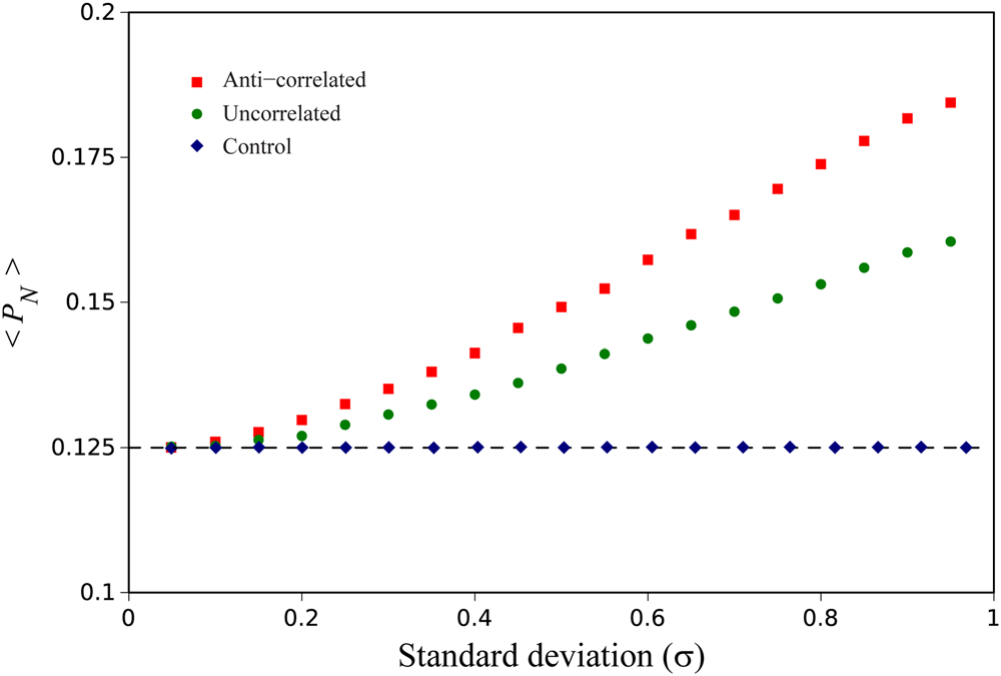
The average mutant fixation probability (starting with one mutant cell) as a function of the standard deviation of the bimodal fitness distribution for different degrees of correlation between mutant and non-mutant fitness values (*N* = 8), obtained from the exact analytical calculations for the death-birth Moran process (see the Method Section).

Here we model GEI using a Moran process with two alleles, a pre-existing allele “A”, and a newly arising mutation “a”, where we randomly assign fitness values based on genotype and position in the ring. We consider three such GEI models. In the first, fitness is purely a function of location in the ring, with the two alleles having equal fitness at any given location. This treatment serves as a control in our numerical results, since the variance in fitness between locations in the ring can be held the same as our other models. In the second anti-correlated selection coefficient model, the selective coefficient for the wild type A allele randomly varies around the ring and is given by *r* + *σ*_*i*_, where *σ*_*i*_ is randomly drawn from a zero mean distribution; while the fitness of the newly arising, mutant allele a at any given location is simply *r* − *σ*_*i*_. Finally, under a third more general GEI model the selective coefficients for the two alleles at each location are either randomly drawn from the same zero mean distribution or the fitness values for A are just shuffled with respect to location for a (in this second case all moments are identical). We are interested in the expected probability of the newly arising mutation fixing, averaged over all realizations of the fitness landscape. As demonstrated in Fig. 1 we observe that the mean fixation probabilities are 1/*N* for a newly arising mutation under our control condition, whereas under our other two models, where GEI exists, newly arising mutations appear to have a selective advantage and fix with a probability greater than 1/*N*. The increased likelihood of fixation cannot be explained simply by a reduction in effective population size in the population due to a high variance in reproductive success^49^, since the controls have an identical variance in fitness yet do not exhibit increased fixation rates.

**Figure 2:**
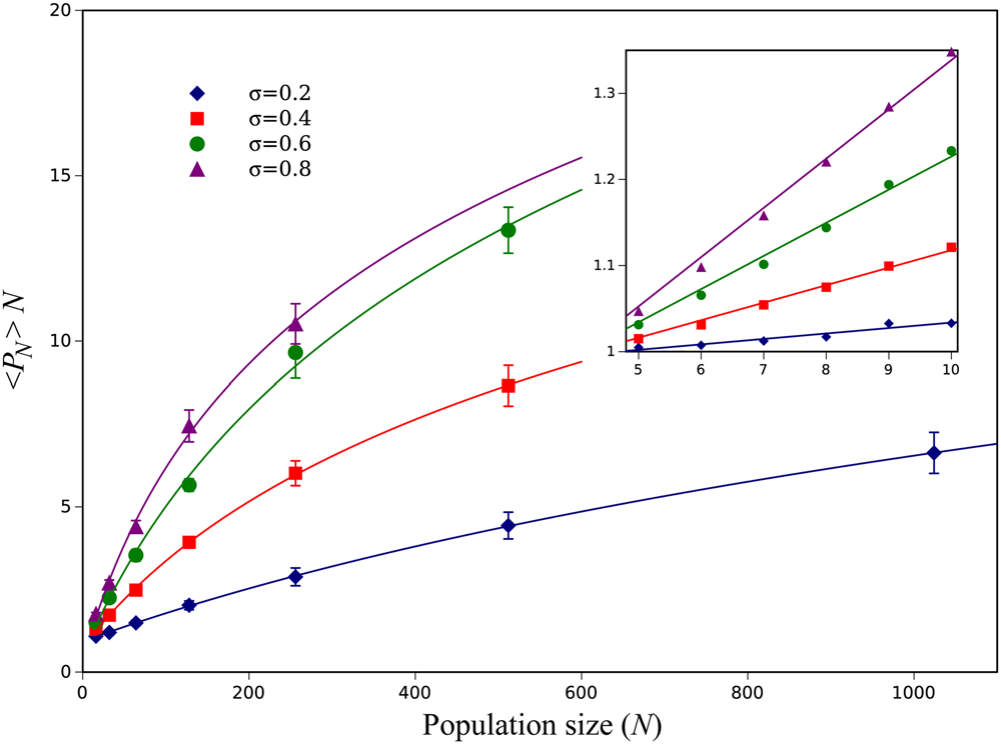
The average mutant fixation probability times *N* as a function of *N* for a death-birth Moran process in a circle. The fitness of both mutants and wild types comes from the same probability distribution and is given by 1 − *σ* and 1 + *σ* with equal probability (the bimodal distribution). For a neutral mutant, this function is expected to look like a horizontal line at level 1; a deviation from horizontal indicates selective advantage. The inset shows the behavior for small values of *N*. The points in the main figure as well in the small internal figure are based on stochastic simulations and error bars are standard error of the mean for a set of 5 realizations. The solid curves in the main figure are based on fitting the numerical simulation data to logarithmic functions. In the small internal figure, curves show the results of exact analytic calculation.

Under our GEI Moran model, newly arising mutants with no average selective advantage behave as if they are advantageous. For varying values of *N*, we carried out numerical simulations of the Moran model and verified results by solving the corresponding Kolmogorov system of equations (see the Method Section). The results for a circle are presented in Fig. 2 where the average fixation probability of a newly arising mutation (〈*P*_*N*_〉, where 〈.〉 is the expected value with respect to a given probability distribution) times *N* is plotted against *N* (under neutrality 〈*P*_*N*_〉*N* = 1 so this is a convenient way to represent the increased fixation probability). For relatively small values of *N*, 〈*P*_*N*_〉*N* increases linearly with N, while the increase of 〈*P*_*N*_〉*N* appears to become sub-linear as*N* becomes large. The magnitude of the effect is large and depends on the variance and the higher moments of the underlying fitness distribution with respect to location. While the increased fixation probability is only about 1% for *N* = 4, fixation probabilities can increase by a factor of twenty for larger *N*. The larger the variance in fitness across environments the greater the deviation of *P*_*N*_ from the neutral result for small values of *N*, but at the same time the lower the size *N* at which the behavior of 〈*P*_*N*_〉*N* becomes sub-linear.

The linear regime (see the panel in Fig. 2) allows us to calculate the effective fitness increment acquired by the non-favored minority allele. In the simple (non-random) Moran process on a complete graph (where all cells are connected), fixation probability of an advantageous mutant with fitness *r* (with wild type fitness given by 1) is given by (1 − 1/*r*)/(1 − 1/*r* ^*N*^) ≈ (*r* − 1)/*r* (for large *N*). Similarly, for a circle this quantity is given by 2(*r* − 1)/(3*r* − 1). It follows that fixation probability times *N* will behave as a linear function in *N*. Finding the slope of straight lines in the panel of Fig. 2 (for circles) and Supplement Figure S1 (for complete graphs) and calculating the effective fitness of the mutants, we can estimate the amount of positive selection experienced by minority mutants in each case. The results are presented in Supplement Figure S2. As expected, the fitness increment is zero for *σ* = 0 and it grows with standard deviation, reaching an 8% fitness increase as the standard deviation approaches 1.

We have extended our calculations to the birth-death formulation of the Moran process and to the Wright-Fisher (diploid) model (see Supplement). Again, the minority mutant in the presence of randomness behaves as if it is selected for. The magnitude of the effect is larger for the birth-death process compared to the death-birth process, and even larger for the Wright-Fisher model. For these two models, the mutant fixation probability increases above 1/*N* even in the *N* = 3 case (Supplement Figures S3 and S4). The case *N* = 3 in the death-birth Moran process is special, because following a death event, only two cells remain to compete with each other for the chance of reproduction. As a consequence, the *N* = 3 case for the death-birth process behaves similarly to the *N* = 2 case for the other two processes (birth-death Moran and Wright Fisher), exhibiting the probability of fixation of 1/*N*. Moreover, we have calculated the fixation probability for a 2D lattice (Supplement Figures S5) and for different shapes of the probability distributions (Supplement Figures S6 and S7). The same core results hold in all these situations.

As the fitness probability distributions of mutants and wild type cells are identical, the only factor that differentiates newly arising from pre-existing alleles under our model is the fact that initially newly arising mutations are a minority. In fact, newly arising mutations fix at a rate greater than that expected under neutrality only if initially they are minority. We carried out numerical simulations where we started the newly arising mutation at frequencies greater than 1/*N* and looked at fixation probabilities relative to the same probability expected under neutrality (see Supplement Figure S8). It appears that the increased likelihood of fixation under our model is primarily due to initially rare variants being much more likely to enter the population, and as the new mutation become more common its behavior is more neutral like. It is of interest that once the new mutation reaches a frequency of greater than 50% its likelihood of eventual fixation is actually lower than under neutrality, this makes intuitive sense since the pre-existing allele at the locus then becomes the rarer more favored allele. The “advantage” afforded by being a minority can override a reproductive disadvantage.

**Figure 3:**
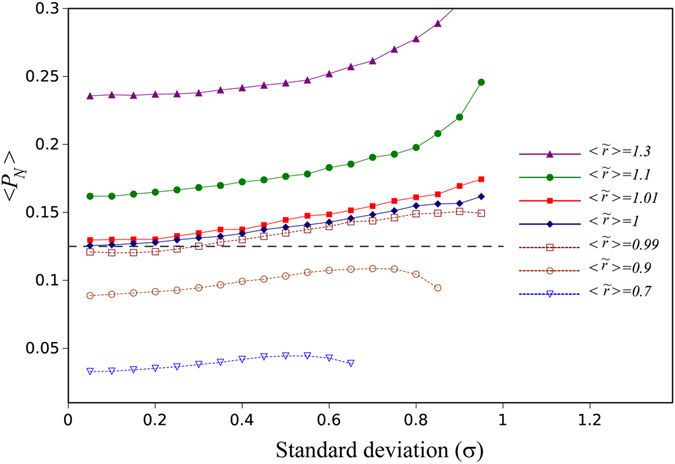
The average mutant fixation probability as a function of the standard deviation of the bimodal fitness distribution (*N* = 8) for different average fitness values of the mutants, with 〈*r*〉 = 1 and $\sigma =\mathop{\sigma }\limits^{ \sim }\,\,({\rm{e}}{\rm{q}}{\rm{u}}{\rm{a}}{\rm{l}}\,{\rm{s}}{\rm{t}}{\rm{a}}{\rm{n}}{\rm{d}}{\rm{a}}{\rm{r}}{\rm{d}}\,$ ${\rm{d}}{\rm{e}}{\rm{v}}{\rm{i}}{\rm{a}}{\rm{t}}{\rm{i}}{\rm{o}}{\rm{n}}{\rm{s}}),\,{\rm{i}}{\rm{n}}\,\,{\rm{a}}\,{\rm{d}}{\rm{e}}{\rm{a}}{\rm{t}}{\rm{h}}-{\rm{birth}}\,{\rm{Moran}}\,{\rm{process}}$. Normally, a neutral mutant will have 〈*P*_*N*_〉 = 1/*N* = 0.125 (dashed horizontal line). Mutants with the mean fitness less than that of the wild type behave as if they are advantageous.

Some intuition that contributes to our understanding of this interesting phenomenon may come from considering different configurations of random landscapes and thinking about resulting fixation probabilities. For a minority mutant (say, one mutant), the biggest hurdle to overcome is to not go extinct and expand to a sizable community. Once this is achieved, the probability of extinction becomes small, and the probability of fixation only weakly depends on *N*. Now, suppose that *N* is large, and consider all fitness configurations in a vicinity of size M around the mutant. A certain fraction of configurations (which is independent of *N*) will be favorable, that is, in all the spots inside the vicinity the mutant will have higher fitness than the wild type; the probability of this is calculated easily from combinatorics, and depends on *M* but not on *N*. When this happens, the mutant will rise with a high probability to size M, and therefore will have completed the hardest part of the journey. What is left is to fixate from size M, which is done with a probability that decays with *N* slower than 1/*N*. This shows that because of the presence of favorable fitness landscapes, a minority mutant receives a boost in the probability of fixation, which only slowly decays with *N*. As a result, its fixation probability is higher than neutral. Figure 3 demonstrates that mutants whose fitness values come from a distribution with a lower mean could behave as if they are advantageous (that is, fixate with a probability larger than1/*N*). This shows that being a minority can compensate for having a lower mean fitness.

**Figure 4:**
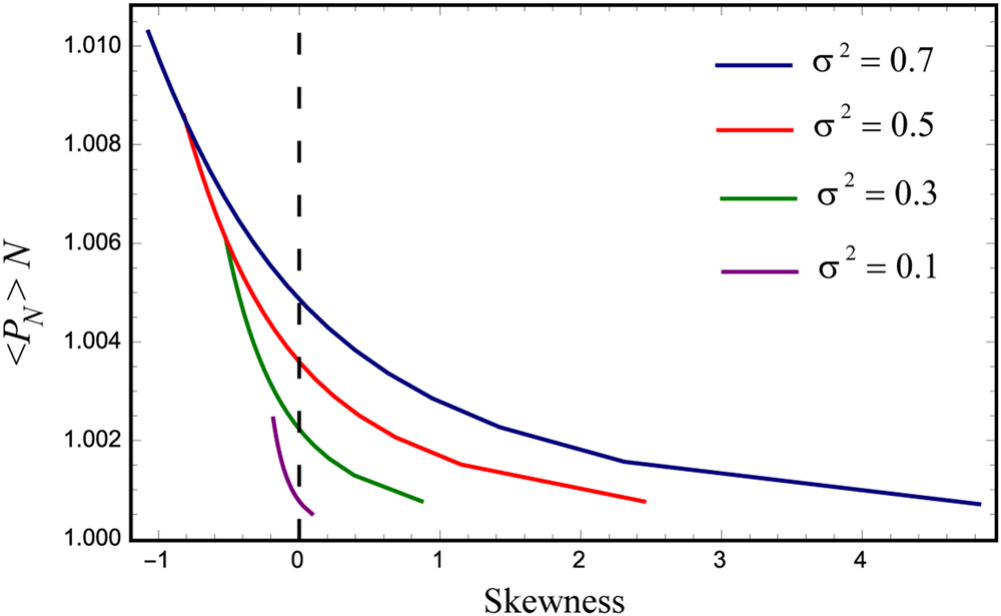
The function 〈*P*_*N*_〉*N* in terms of the skewness (*N* = 4, death–birthMoranprocess). We assume that the mean fitness of the mutants is equal to that of the wild types, and $\sigma =\tilde{\sigma }$. As before, 〈*P*_*N*_〉*N* greater that unity indicates that the mutants experience selective advantage; the effect is the largest for the largest negative skewness.

**Figure 5:**
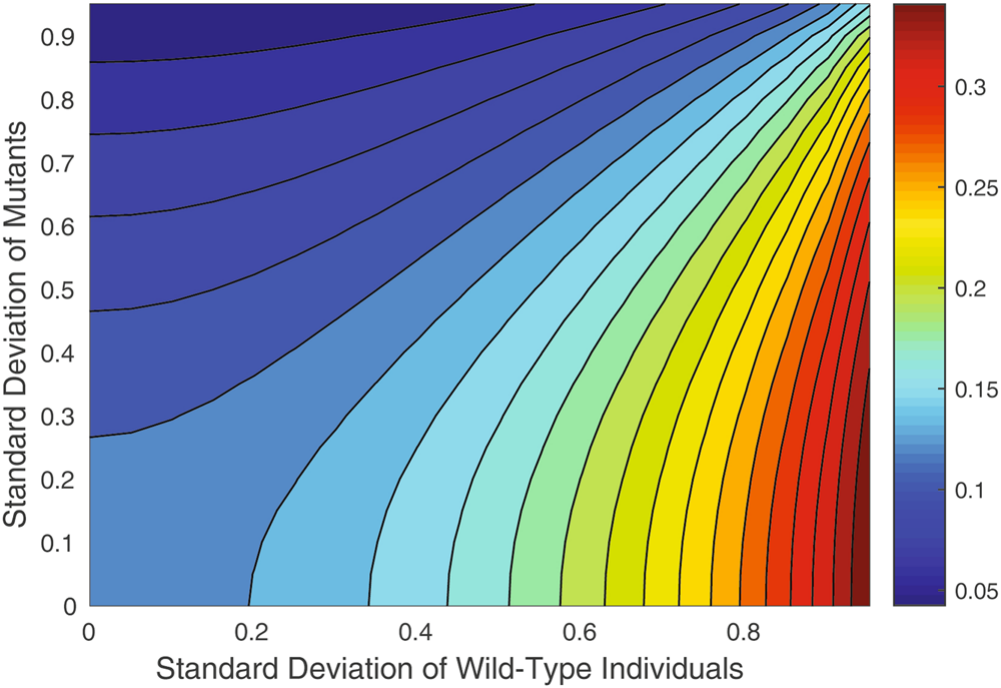
The heat plot for *P*_*N*_ as a function of the two standard deviations (*N* = 8, death–birthMoranprocess). The amount of variance in the distribution of fitness values of the wild types (mutants) increases along the horizontal (vertical) axis. The largest fitness advantage of mutants (in red, right bottom corner) corresponds to the largest variation of the wild types and smallest variation of the mutants.

If we keep both the mean and the standard deviation of the fitness distributions constant, and vary the third moment (skewness), we observe that 〈*P*_*N*_〉*N* is the largest for the largest negative skewness (Fig. 4). Next, let us suppose that while having equal means, the standard deviation of the two distributions can be different (where larger standard deviations signify larger randomness). It turns out that for the minority population, it is beneficial to be as deterministic as possible. Larger standard deviations of the background distribution and smaller standard deviations of the mutant distribution lead to higher values of mutant fixation probabilities (Fig. 5).

The effects of spatial structure and heterogeneity are important in biological models, including evolutionary, biomedical, and social network models. It is known that structure of the network can suppress or amplify the fixation probability^50, 51^. In addition, the heterogeneity of the spatial structure has impacts on the fixation probability, see for example^21, 44, 48, 52, 53^. Although several papers have focused on the study of heterogeneous networks, less effort has been devoted to understanding the effect of heterogeneity, due to the spatial fitness distribution and environmental stress, and its effect on the invasion probability. In real systems, the fitness of individuals strongly depends on the microenvironmental conditions. For example, in models of bacterial growth or cancer progression, fitness can be a function of the spatial distribution of nutrients and microenvironment. As demonstrated here, random fitness distributions can significantly influence biological and social systems dynamics, leading to an increased probability of newly arising mutations fixing.

Although the model of this paper is motivated in terms of the fixation probability of a newly arising mutation in a haploid population, it is equally applicable to a clone that is reproducing and colonizing new environments. Thus, an important biological application of the ring geometry studied here is the model of a human colonic crypt, where stem cells are situated along circular bands (in this context, fixation is referred to as monoclonal conversion)^54^. These cells divide leading to proliferation or differentiation (equivalent to removal in our models), and the origins of colon cancer can be studied by examining selection dynamics of mutants in such a system. Of particular importance is the*APC* ^+/−^ mutant, which in many models is considered neutral or slightly disadvantageous. Such a mutant taking over in one of about 10^7^ crypts in a colon is often a first step in the pathway to colon cancer. If the randomness of the environment is taken into account, the theoretical likelihood of such an event can be significantly higher than predicted by the standard theories. In fact, given the relatively small population size of the stem cell pool, such mutants will behave as if they are advantageous, leading to very different dynamics, see for example^16, 55^.

The results of our model have far reaching consequences: the rate of molecular evolution may be higher for genes that are important in GEI, the relationship between patterns of standing variation versus divergence may be more complex than is generally thought, and in processes where fixation events are commonly observed (e.g., tumorigenesis)–fixed mutants may not necessarily be “adaptive”, but instead only exhibit environment heterogeneity. A large part of population genetics theory relies on the idea that neutral alleles fix with probability 1/*N*_*e*_. This fundamental notion plays a role in famous concepts such as Kimura’s molecular clock, genetic drift, evolutionary divergence, and coalescence^56, 57^. In this paper we show that non-favored mutants can behave as if they are selected for, and fixate at rates much higher than 1/*N*_*e*_ under models where genotype by environmental interactions exist. Our findings have implications for studies of between species DNA divergence and within species standing variation, which may not match neutral expectation. Tests for neutrality versus selection, as well as inferences about demography will also be impacted^58^.

## Electronic supplementary material

Supplementary Information
