# Supplementary material for: Genotype by random environmental interactions gives an advantage to non-favored minor alleles
Source: Sci Rep. 2017 Jul 12;7:5193. doi: 10.1038/s41598-017-05375-0 (PMC5507875; doi:10.1038/s41598-017-05375-0)
Supplement: Supplementary file 1 — Supplementary Information [file 41598_2017_5375_MOESM1_ESM.pdf]

Genotype by random environmental interactions gives an advantage to non-favored minor alleles

A. Mahdipour–Shirayeh [1], A. H. Darooneh [2], A. D. Long [3], N. L. Komarova [4,*], M. Kohandel [1,*]

Supplement Figures S1 and S2

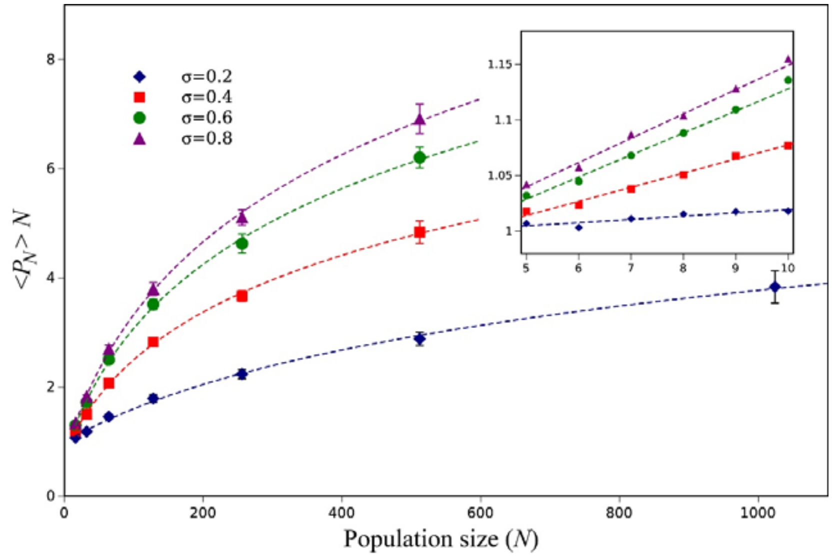

FIG. S1: The average mutant fixation probability (starting with one mutant cell) times N in terms of the standard deviation of the bimodal fitness distribution for a complete graph. Calculations are performed for the death-birth Moran process. The points in the main figure as well in the small internal figure are based on stochastic simulations and error bars are standard error of the mean for a set of 5 realizations. The solid curves in the main figure are based on fitting the numerical simulation data to logarithmic functions. In the small internal figure, curves show the results of exact analytic calculation.

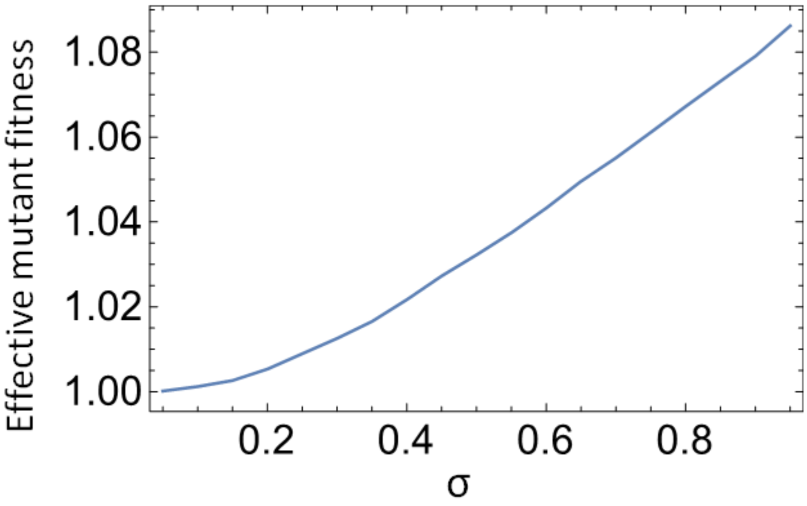

FIG. S2: The effective fitness of the mutants as a function of the standard deviation of the bimodal fitness distribution. The calculations are performed for the death-birth Moran process and are based on data in the panel of figure 2 (for circles) and figure S3 (for complete graphs).

Moran process on a complete graph

Consider a set of N spots that are characterized with random (between realizations), constant (throughout a given realization) fitness values, which we denote $a_1, a_2, \cdots, a_N$ for wild type cells and $b, b_2, \cdots, b_N$ for mutant cells. The iterations proceed as follows. Suppose the current state is given by a binary sequence

$$S = (s_1, s_2, \cdots, s_N) \ , \ \ s_i \in \{0,1\}$$

where 1 stands for a mutant and 0 stands for a wild type cell. Let us denote the probability of fixation starting from state S as P_S. These probabilities satisfy the system

$$P_S = \sum_T P_{S \to T} \, P_T \qquad (1)$$

where S and T are all binary sequences of length N, and the boundary conditions are

$$P_S = \begin{cases} 0 & : \quad S = (0, \cdots, 0) \\ 1 & : \quad S = (1, \cdots, 1) \end{cases}$$

To set up the transition matrix, suppose that the system state after the update is given by a binary sequence

$$T = (t_1, t_2, \cdots, t_N) \ , \ \ t_i \in \{0,1\}$$

Not all transitions are possible in a single update of the Moran process. A transition can happen from generation S to generation T only if the Manhattan distance between them satisfies $|S - T| \leq 1$.

Transition probabilities between states that satisfy this inequality can be calculated, and they are different depending on the order of the birth and death events in the Moran updates. Below we examine the two implementations of the Moran process, the death-birth and the birth-death processes.

The death-birth process. In this formulation, we first eliminate a cell, such that each cell has the same probability $1/N$ to die. Then another cell proliferates, with the probability proportional to its fitness. If the number of mutants increases at location k, that is, if $t_k - s_k = 1$, then a death of a wild type cell at location k is followed by a division of a mutant cell, and

$$P_{S\to T} = P_{db}^{\uparrow} = \frac{1}{N}\frac{\sum_{i=1}^{N} s_i b_i}{\sum_{i\neq k}(s_i b_i + (1-s_i)a_i)}$$

where the superscript stands for the increase in mutant number, and the subscript for the death-birth process. If the number of mutants increases at location k, that is, if $t_k - s_k = -1$, then a death of a mutant cell at location k is followed by a division of a wild type cell, and

$$P_{S\to T} = P_{db}^{\downarrow} = \frac{1}{N}\frac{\sum_{i=1}^{N} (1-s_i)a_i}{\sum_{i\neq k}(s_i b_i + (1-s_i)a_i)}$$

Finally, the probability of no change is given by

$$P_{S\to S} = 1 - P_{db}^{\uparrow} - P_{db}^{\downarrow}$$

The birth-death process. In this formulation, we first pick a cell for division (again, this is based on the cell's fitness), and then this is followed by a death of a randomly (uniformly) chosen cell, after which the progeny of the cell that divided replaces the dead cell. We assume that a cell that just divided cannot die in the same update. If the number of mutants increases at location k, then a death of a wild type cell is followed by a division of a mutant cell, and

$$P_{S\to T} = P_{bd}^{\uparrow} = \frac{1}{N-1}\frac{\sum_{i=1}^{N} s_i b_i}{\sum_{i=1}^{N}(s_i b_i + (1-s_i)a_i)}$$

If the number of mutants increases at location k, then a death of a mutant cell is followed by a division of a wild type cell, and

$$P_{S\to T} = P_{bd}^{\downarrow} = \frac{1}{N-1}\frac{\sum_{i=1}^{N}(1-s_i)a_i}{\sum_{i=1}^{N}(s_i b_i + (1-s_i)a_i)}$$

As before, the probability of no change is given by

$$P_{S\to S} = 1 - P_{bd}^{\uparrow} - P_{bd}^{\downarrow}.$$

There are two differences between the transition probabilities in the death-birth and the birth-death processes. The first difference is the factor $1/(N-1)$ instead of a factor $1/N$, accounting for the fact that in the birth-death process, a newly divided cell cannot be picked for death. It turns out that this factor is unimportant, because it cancels out from equation (1). The second difference is that in the death-birth process, the cell at site k (where the change occurs) does not participate in divisions (because it has died), and in the birth-death process it does. Mathematically it is reflected in the exclusion of the site k from the summation in the probability expressions for the death-birth process. It turns out that this makes a significant difference in the behavior of the system.

The behavior of the two implementations of the Moran process is illustrated in Figure S1. There, the probability of mutant fixation is plotted against standard deviation for both processes, for three different population sizes. One can see that the increase in the fixation probability is larger for the birth-death process. Interestingly, for the birth-death process, one observes an increase in the fixation probability even for $N = 3$ (recall that in death-birth process the probability of fixation for $N = 3$ is a constant given by $1/N$).

Supplement Figures S3

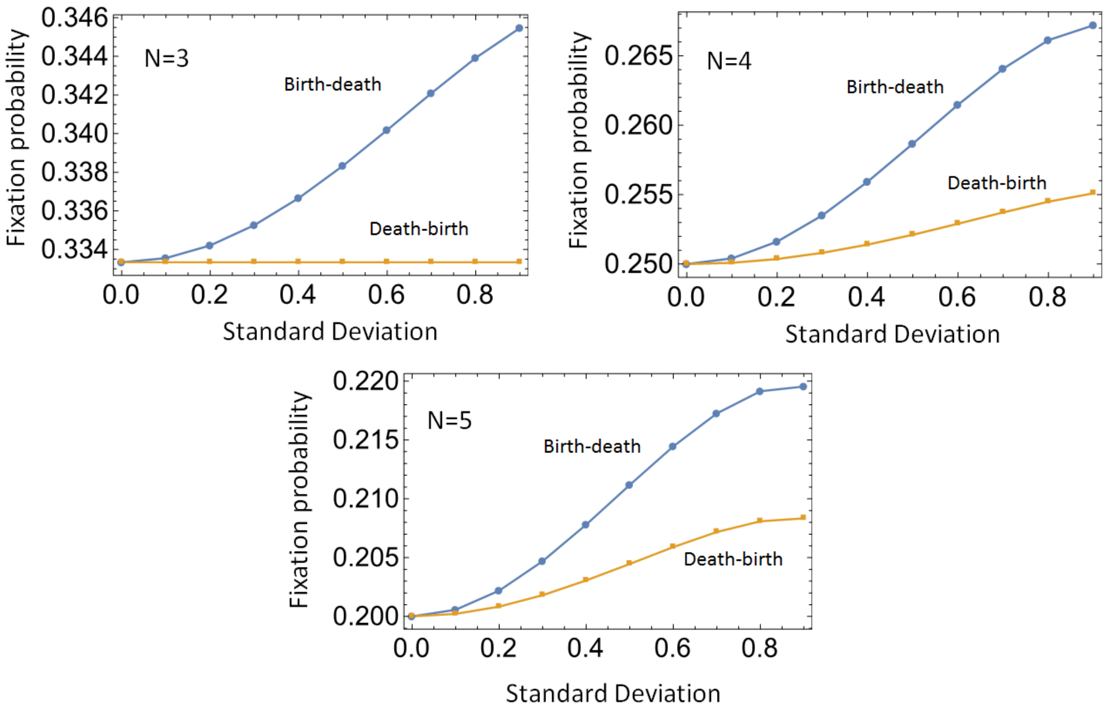

FIG. S3: The average mutant fixation probability (starting from one cell) in terms of the standard deviation of the bimodal fitness distribution on a complete graph for $N = 3, 4, 5$, for the two implementations of the Moran process: death-birth and birth-death.

Haploid Wright-Fisher model

Consider a haploid Wright-Fisher model, where again there is N spots that are characterized with random (between realizations), constant (throughout a given realization) fitness values, which we denote $a_1, a_2, \cdots, a_N$ for wild type cells and $b, b_2, \cdots, b_N$ for mutant cells. The iterations proceed as follows. The next generation is populated by randomly drawing (with replacement) cell types (mutant or wild type) from the current population. As before, let us suppose that the current generation is given by a binary sequence

$$S = (s_1, s_2, \cdots, s_N) \ , \ \ s_i \in \{0,1\}$$

Then the probability to pick a mutant is given by

$$q = q_{\text{hapl}} = \frac{\sum_{i=1}^{N} s_i b_i}{\sum_{i=1}^{N} (s_i b_i + (1 - s_i) a_i)}$$

Here, the subscript stands for “haploid”. This formula reflects the fact that the probability for an individual to be picked is proportional to its fitness, and fitness is defined by the location (and the corresponding random fitness value). The probability to create the next generation given by a binary sequence

$$T = (t_1, t_2, \cdots, t_N) \ , \ \ t_i \in \{0,1\}$$

is calculated as

$$P_{S \to T} = \prod_{i=1}^{N} q^{t_i} (1-q)^{1-t_i}$$

Let us denote the probability of fixation starting from state S as P_S. These probabilities satisfy the system

$$P_S = \sum_T P_{S \to T} \, P_T$$

where S and T are all binary sequences of length N, and the boundary conditions are

$$P_S = \begin{cases} 0 & : \quad S = (0, \cdots, 0) \\ 1 & : \quad S = (1, \cdots, 1) \end{cases}$$

It is easy to show that in the $N = 2$ case, the probability of mutant fixation is equal to $1/N = 1/2$. Figure S2 illustrates the behavior of this model for $N > 2$. The probability of mutant fixation is plotted as a function of the standard deviation (σ) for $N = 3, 4, 5$. We can see that for $\sigma = 0$, the fixation probability is given by $1/N$, and it grows with standard deviation, even for the $N = 3$ case.

Supplement Figures S4

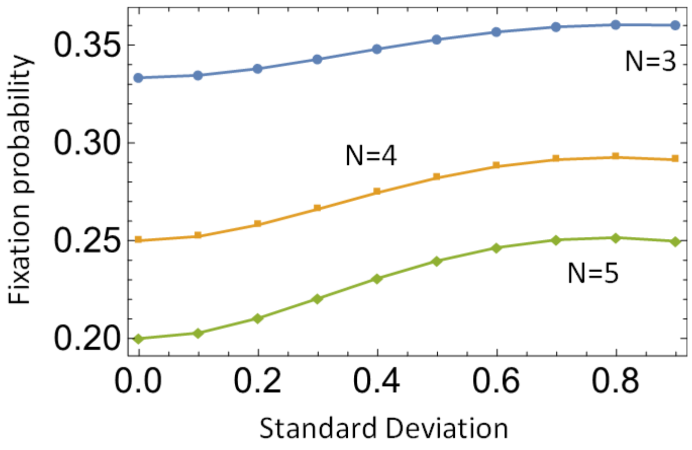

FIG. S4: The average mutant fixation probability in terms of the standard deviation of the bimodal fitness distribution for the haploid Wright-Fisher model for $N = 3, 3, 5$.

Supplement Figures S5 - S8

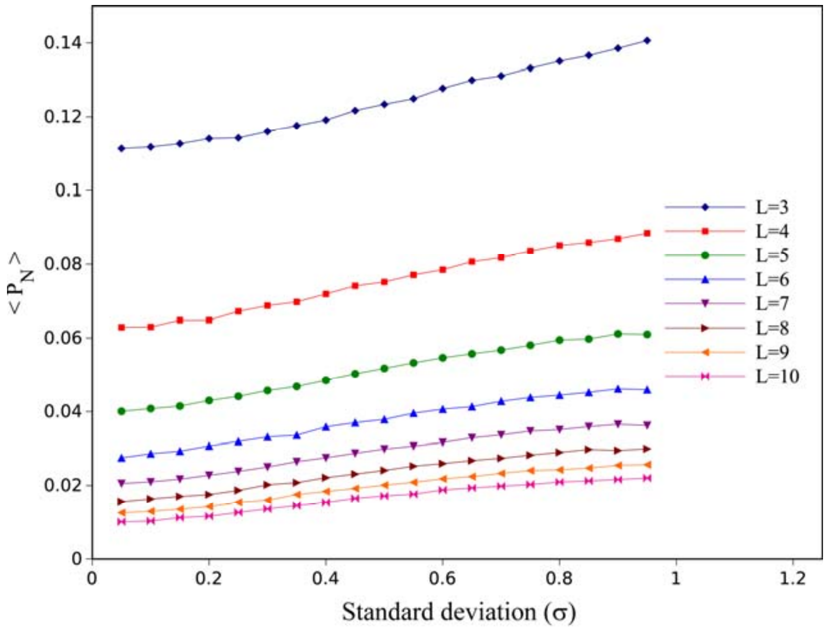

FIG. S5: The average mutant fixation probability in terms of the standard deviation of the bimodal fitness distribution for a 2D lattice $L \times L$, in the death $-$ birth Moran process.

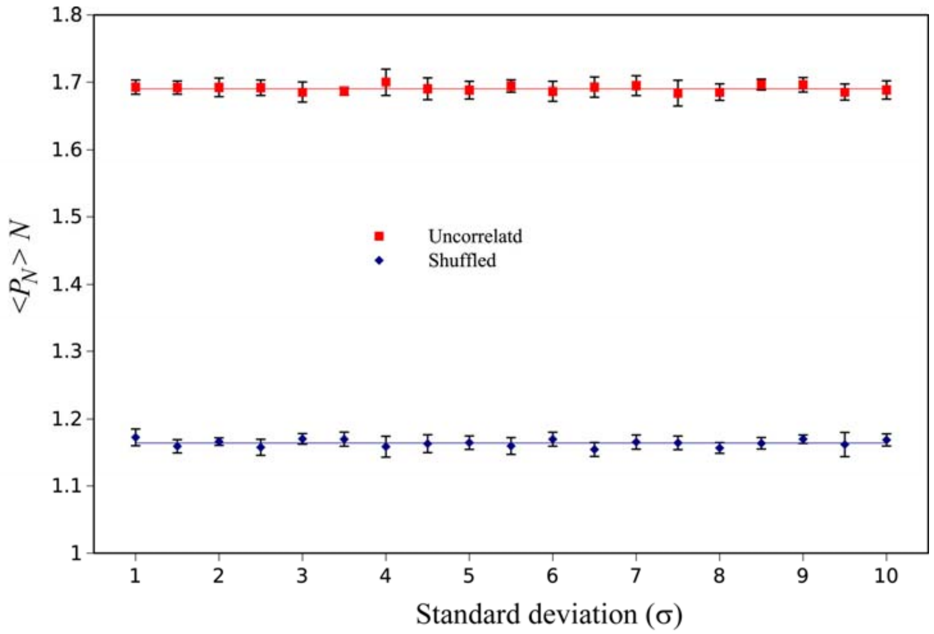

FIG. S6: The average mutant fixation probability times N in terms of the mean (or standard deviation) of the exponential distribution (death-birth Moran process). The fitnesses at each location are either randomly drawn from the same unit mean distribution (uncorrelated) or the fitnesses for A are just shuffled with respect to location for a (shuffled). In both cases, the fixation probability is greater than $1/N$. In the shuffled case, we pick N random fitness values and assign them to the wild type. Then we shuffle these fitness values and assign them to the mutants. Thus the fitness values for the mutants are the same shuffled N values. In the uncorrelated case, the fitness of both wild type individuals and mutants are chosen separately at random. Note that in this case there is no dependence on standard deviation, because for the exponential distribution, the standard deviation is not independent of the mean value.

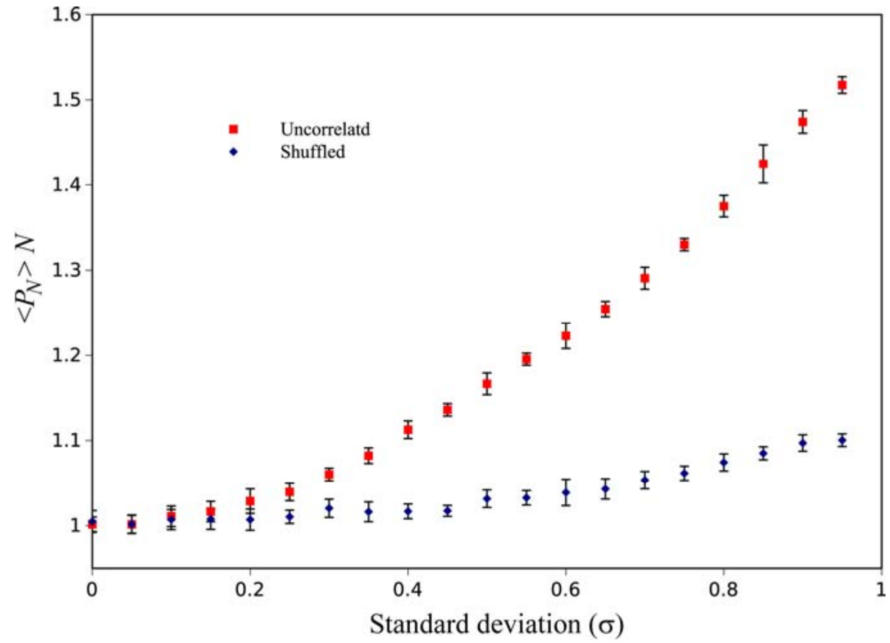

FIG. S7: The average mutant fixation probability times N in terms of the standard deviation of the uniform distribution with the mean $\mu = \sqrt{3}$ (death-birth Moran process). The shuffled and uncorrelated cases are introduced in the previous figure; the difference in results for these two cases can be explained as follows. In the shuffled case, the mutant and wild type fitness values are not independent (the number of $1 - \sigma$ values and $1 + \sigma$ values is preserved). For example, in the special case where all wild type fitness values happen to be $1 - \sigma,$ the mutant fitness values will also be $1 - \sigma$ by construction. The largest effect of randomness on the fixation probability of mutants is observed when the fitness values are anti-correlated (FIG. 1), and the zero effect is observed when they are completely correlated. The uncorrelated case (independent fitness values) is somewhere between these two extreme scenarios. The shuffled case is then expected to be between the correlated and uncorrelated cases. The effect of randomness there is smaller compared to the uncorrelated case, as seen in this figure.

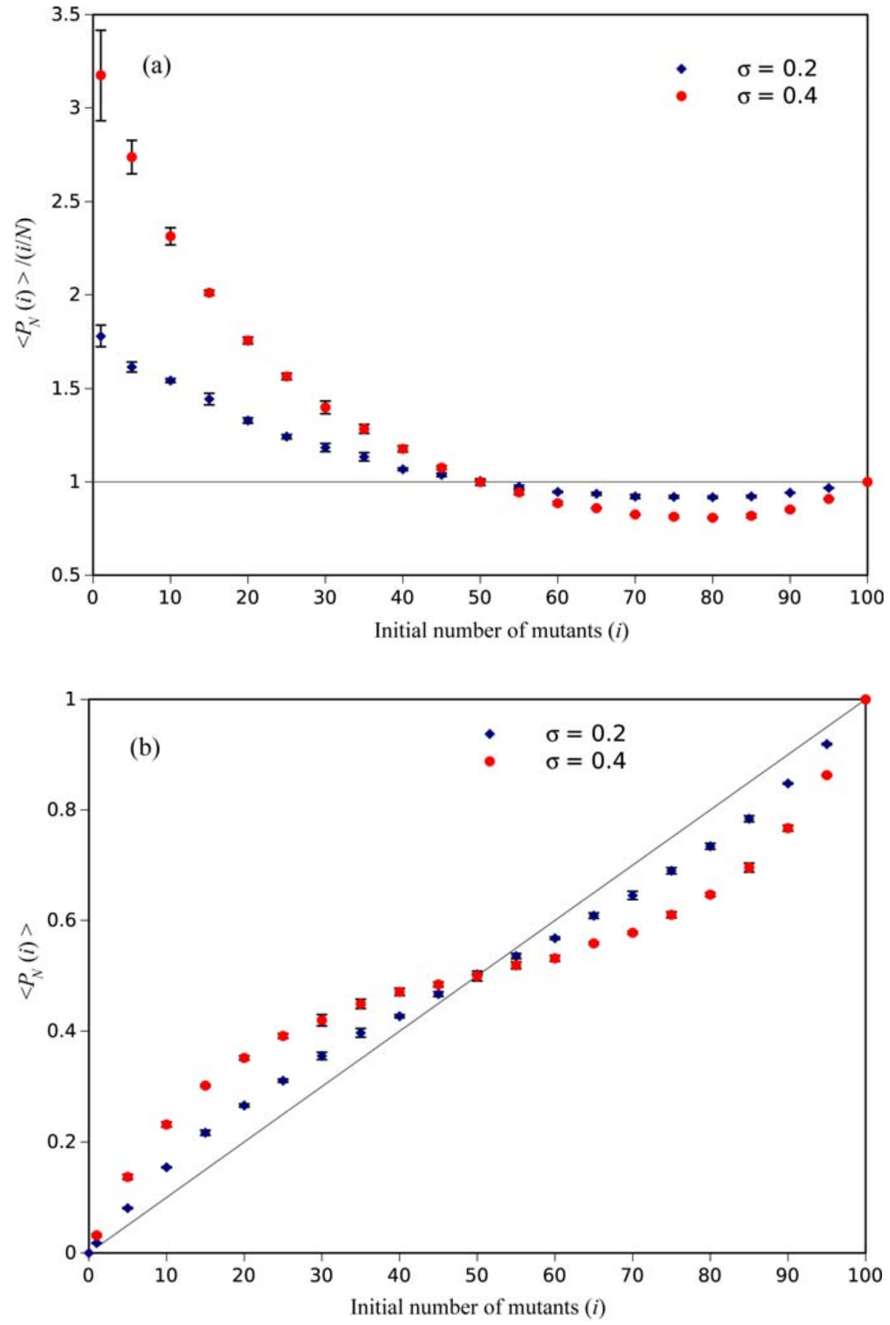

FIG. S8: (a) The average mutant fixation probability over the expected probability under neutrality (i/N), and (b) the average fixation probability, in terms of initial number of mutants (for the bimodal fitness distribution). Calculations are performed for the death-birth Moran process.
